# Is It Time for Operation Warp Speed in Transplant Research?

**DOI:** 10.1097/TXD.0000000000001073

**Published:** 2020-10-20

**Authors:** Roslyn B. Mannon

**Affiliations:** 1 Division of Nephrology, Department of Medicine, University of Nebraska Medical Center, Nebraska-Western Iowa VA Medical Center, Omaha, NE.

Academic medicine has undergone a gender transformation over the last 3 decades, with women now making up the majority of medical students trained in the United States and traditionally male-dominated specialties like Internal Medicine and General Surgery composed of ≈40% women. Yet, there continues to be a remarkable disconnect in terms of career advancement, with women representing only 41% of faculty members in academic medical centers in the United States and only 18% of leadership positions including department chairs and deans headed by women.^1^ These numbers are even more dismal when you recognize that only 13% of full-time faculty are underrepresented minorities. Likewise, trainees in doctorate programs in 2017 were 60% women, but only 46% women make up postdoctoral positions in the United States and only about a third of basic science teaching faculty composed of women. Although these numbers have improved over the last decade, the changes are incremental at best. Key takeaways from this Association of American Medical Colleges report,^1^ which includes not only medical training but doctoral and postdoctoral experiences, are that institutions “must continue to mentor women” and “to encourage them to pursue these advanced positions and promote careers in academic medicine.” In short, this is great advice but difficult to find a one-size-fits-all solution when the challenges here are more complex. They include work-life balance, sexual harassment, implicit bias, pay gap, and academic productivity^2^ and vary by specific center and geography. Although these comments reflect medical training and careers in general, to date, there have been deficiencies identified in transplantation research involving 2 different continents.^3,4^

Key for the career advancement of promotion and tenure is individual productivity, typically defined as grant funding and peer-reviewed publications. The latter are also considered as a metric of an individual’s impact on their field. Some academic centers utilize automated databases, such as Scopus, to curate and regularly update bibliographies of faculty members, highlighting expertise and enhancing reputation.^5^ Moving more toward author-specific metric, the Hirsch or h-index, a measure that combines papers (quantity) and citations (ie, quality or impact) that is not affected by infrequently cited papers or by increases in citations to already highly cited papers, is frequently used to provide a metric of publication impact by individuals and their future potential as key contributors.^6^ Although not perfect, such tools have become widely accepted and frequently included in reviews of faculty members for promotion. However, a detailed, worldwide analysis of publication metrics for investigators in transplant research has not been previously undertaken.

In this issue of *Transplantation Direct*, Benjamens et al, leveraging research methods used to identify research funding deficiencies in the United Kingdom for transplantation-related research,^3^ evaluate the publication records of female and male investigators in transplantation.^7^ Not surprisingly, of nearly 16 000 publications eligible for the study in first quartile “high impact” journals for each field in transplant research, woman comprised only 36% of first authors and only 30% of senior authors. Although this represents an increase over the last 2 decades, this disparity was apparent in nearly all of the top 10 scientifically productive countries in transplant research. Most extreme was Japan, where only 18% of women were first authors, whereas the Netherlands demonstrated the least disparity.These findings were accompanied by statistically lower citations for both female first authors and senior authors versus male. The distribution of highly cited (and “older”) publications was also skewed, with male dominance in papers cited more than 200 times, both as first and senior authors. There was an accompanying striking disparity in research funding cited in these publications, again with women significantly lower at 42% compared with men at 58%. The authors conclude that the gender disparity in academic research exists in transplantation research, suggesting an “active approach to eliminating potential bias in research reporting and funding rewarding.”

Although this report has intrinsic limitations, including exclusion of ≈17 300 papers without first author names or because of the inability to determine gender based on name, the focus on the top 10 research-productive countries (although 88 included in the analysis), and citation number, which is dependent on time following publication, the findings comprise a large data set and indicate that even in technically advanced countries, these disparities continue. One only can imagine the further hurdles in more patriarchal societies and those with less scientific advancements.

Kudos to COVID-19 to shed light (again) on structural racism and chauvinism that is present not only in healthcare, but academia. Women are publishing less during the pandemic, including on preprint platforms, and are registering fewer research projects since the pandemic started.^8^ There are several inferred reasons including the expanded responsibilities of family care on women compared with men, coupled with ongoing professional responsibilities such as teaching and clinical care that many senior faculty (ie, male counterparts) may not have to address. Women, in some instances, have no choice but defer their academic careers, and the impact and outcomes are only starting to be recognized.

Can a broken system be fixed? Admittedly, in an academic career, responsibility lies with the individual, but also in the mentorship of the individual. In this regard, the Transplantation Society made a critical commitment to women investigators and clinicians, even before published data. When elected to the presidency of the Transplantation Society in 2004, Dr Kathryn Wood became the first woman to achieve that office and immediately created the Women in Transplantation initiative. Although initially focused on mentorship across the continents, the strategic “reboot” supported by President Nancy Ascher in 2017 led to the creation of Pillar 1, focused on networking and career advancement, and Pillar 2, supporting studies in sex and gender in transplantation. Recent “crowd-sourced” publications^9,10^ highlight the imperatives to study sex and gender in immunology and clinical care and outcomes. Also during this period of awareness, there have been calls to action by the Transplantation Society of Australia and New Zealand^4^ and nephrology community^11^ for opportunities for women leaders. Only through frank discussion about work-life balance, academic advancement through peer networks across continents, and self-promotion of female peers’ accomplishments, can change occur. During this most challenging time, a defined path to academic “re-entry” and recognition of the past barriers to success are needed, with an imperative to immediately clear those hurdles. We cannot afford to lose a generation of talented women investigators. We need our own, “Operation Warp Speed.”
